# Antitumor immunity and therapeutic properties of marine seaweeds-derived extracts in the treatment of cancer

**DOI:** 10.1186/s12935-022-02683-y

**Published:** 2022-08-23

**Authors:** Mostafa M. El-Sheekh, Mohamed Nassef, Eman Bases, Shimaa El Shafay, Rania El-shenody

**Affiliations:** 1 Botany Department, Faculty of Science, Tanta University, Tanta, 31527 Egypt; 2 Zoology Department, Faculty of Science, Tanta University, Tanta, 31527 Egypt

**Keywords:** Seaweeds, Immunomodulation, Antitumor immunity, Apoptosis

## Abstract

Marine seaweeds are important sources of drugs with several pharmacological characteristics. The present study aims to evaluate the antitumor and antitumor immunological potentials of the extracts from the brown alga *Padina* *pavonica* and the red alga *Jania* *rubens*, inhibiting the Egyptian marine coasts. Hep-G2 cell lines were used for assessment of the antitumor efficacy of *Padina* *pavonica* and *Jania* *rubens* extracts in vitro, while Ehrlich ascites carcinoma (EAC) cells were applied to gain more antitumor immunity and antitumor insights of *P.* *pavonica* and *J.* *rubens* extracts in vivo. In vitro antitumor potentials of *P.* *pavonica* and *J.* *rubens* extracts were analyzed against human liver cancer Hep-G2 cells by MTT and trypan blue exclusion assays. In vivo antitumor immunological potentials of *P.* *pavonica* and *J.* *rubens* extracts at low, high, and prophylactic doses were analyzed by blood counting and flow cytometry in mice challenged with Ehrlich ascites carcinoma (EAC) cells. In vitro results revealed that *P.* *pavonica* and *J.* *rubens* extracts caused significant decreases in the number and viability of Hep-G2 cells in a dose-dependent manner as compared to untreated Hep-G2 cells or Cisplatin^®^-treated Hep-G2 cells. In vivo findings showed that *P.* *pavonica* and *J.* *rubens* extracts at low, high, and prophylactic doses significantly reduced the number and viability of EAC tumor cells accompanied by increases in EAC apoptosis compared to naïve EAC mouse. Additionally, *P.* *pavonica* and *J.* *rubens* extracts at low and prophylactic doses remarkably increased both the total WBC count and the relative numbers of lymphocytes and decreased the relative numbers of neutrophils and monocytes. Flow cytometric analysis showed that *P.* *pavonica* and *J.* *rubens* extracts at the treatment and the prophylactic doses resulted in a significant increase in the phenotypic expressions of CD4^+^ T, CD8^+^ T, and CD335 cells compared to naïve EAC mouse. Overall, both extracts *P.* *pavonica* and *J.* *rubens* possess potential antitumor and antitumor immunological effects with less toxicity, opening new approaches for further studies of the chemical and biological mechanisms behind these effects.

## Introduction

It is well known that cancer is a serious public health concern worldwide because it is a life-threatening illness. There are six characteristics of cancer cells phenotypes or cancer hallmarks: indefinite proliferation, proliferation environmental independence, apoptosis evasion, angiogenesis, invasion, and metastasis to various body sites [1]. The most popular cancer treatments are surgery, radiotherapy, immunotherapy, hormonal therapy and chemotherapy, which include a variety of drugs that invade the body tissue, affect both cancerous and healthy cells' action mechanisms [2]. Chemotherapies have a number of side effects, including anemia, peripheral neuropathy, hair loss, appetite loss and organ impairment [3]. One of the biggest difficulties in chemotherapy is the tumor resistance or tolerance, which is one of the main challenges in cancer chemotherapy and one of the main reasons for failure. Therefore, there is a continuing need to create innovative, efficient, and cost-effective anticancer drugs [4, 5].

Due to the harmful effects and lack of selectivity of the majority of chemotherapeutics, natural pharmaceuticals are chosen for their acceptance and decreased toxicity [6]. Consequently, there is a growing review on the potential and medicinal characteristics of natural pharmaceuticals in improving anticancer drug therapeutic effects and protecting non-tumor tissues from chemotherapy-induced damage [7]. Several target-specific anticancer medications failed to achieve successful effects elucidating a need to search natural products with multi-target features to attain better outcomes [8]. Actually, multi-targeting efficacy of natural products can inhibit the progress of resistance to their anti-tumor approaches [9].

The administration of natural products in cancer therapy is due to their potential on many molecular mechanisms and pathways. Natural products application can promote apoptosis and cell cycle arrest in cancerous cells to prevent cancer progression. Besides, they down-express tumor-stimulating molecular pathways while they up-express tumor-suppressing factors expression level [10, 11]. Natural products triggering tumor-promoting factors: Nuclear factor kappa B (NF-κB) down-regulation and TGF-β1 expression inhibition to inhibit the cancer-associated fibroblasts transformation into pre-carcinogenic cells. Natural product derivatives help in cancer treatment by decreasing the destructive behavior of tumors by suppressing metastasis by preventing epithelial-to-mesenchymal transition mechanism. These profits of natural products led to the increasing use of natural products in cancer therapy [10, 12].

Despite the fact that cancer has been studied for decades, more research is needed to produce anticancer medications that are extremely effective, have low tolerance, and cause fewer adverse effects. Furthermore, with the continued rise in cancer cases and concerns about toxicity, tumor cell resistance, the formation of secondary malignancies, and the unfavorable side effects seen with synthetic medications in recent decades, there has been a raised interest in using natural products to treat cancer [13].

Seaweeds are an attractive species because they can be a rich source of bioactive compounds, including toxins with potential therapeutic applications [14, 15]. Bioactive compounds extracted from different types of seaweeds exhibited anticancer potentials against human cancers [16]. Furthermore, the brown seaweed sterol hydrocarbon fraction showed dose-dependent anticancer efficacy against a number of cancer cell lines, including the Hep-G2 liver cancer cell line, the MCF-7 breast cancer cell line, the A549 colon cancer cell line, and the A549 ovarian cancer cell line [17]. *Jania* *rubens* (Rhodophyta) methanolic extracts demonstrated anticancer activity against the Molt-4 and Jurkat cell lines (IC_50_ values of 60.25 and 62.5 g/mL, respectively), indicating that it could be a good source of anti-leukemia medicines [18]. By blocking vascular endothelial growth factor (VEGF) and natural killer (NK) cell activation, antitumor chemicals from seaweeds can cause cancer cell death via a variety of signaling pathways, including cell cycle arrest, apoptosis, and anti-angiogenesis [19]. These features of natural seaweed products can help to prevent cancer cells from becoming resistant or tolerating them [20].

Immune regulators produced from seaweed have been reported to stimulate immunological cells and boost the body’s immune function [21, 22]. Some seaweed-derived extracts have been studied in biomedical research for biological activities such as anticancer and immunomodulatory properties [23]. Adjuvants generated from seaweed that were used in cancer immunotherapy found to have good results in terms of targeted immune stimulation [24]. Seaweed extracts may have an immune-modulatory impact, which could enhance their anticancer activity in vivo.

Seaweed extracts have been reported to exhibit a variety of biological actions, including anticoagulant and antiviral properties, as well as roles in immunity enhancement, anti-inflammation, blood cholesterol reduction, antioxidation, improved liver and kidney activity, and gastrointestinal protection [25–28]. Studies on the functional and immunomodulating properties of seaweed extracts have recently been published [29].

The immunomodulatory, antitumor immunological and anticancer activities of natural seaweed products are increasing worldwide because they are cost-effective, safe, and effective. They also contain a large number of phytoconstituents that can be used for the development of new therapeutic entities such as antitumor immunotherapy and antitumor drugs. Therefore, we aimed to study the immunomodulatory, antitumor immunological and antitumor efficacy of methanolic extracts of two seaweeds: *Padina* *pavonica* (Phaeophyceae) and *Jania* *rubens* (Rhodophyta) against Ehrlich Ascites Carcinoma (EAC) bearing mice.

## Materials and methods

### Reagents and antibodies

*P*. *pavonica* and *J.* *rubens* extracts were dissolved in phosphate-buffered saline (PBS) and kept at − 80 °C till further studies. Anti-mouse CD4, anti-mouse CD8, and anti-mouse CD335 (NKp46) monoclonal antibodies were purchased from eBioscience (San Diego, CA, USA). Heat-inactivated fetal bovine serum (FBS) (10% v/v), 2-mM l-glutamine, Penicillin/Streptomycin solution (100 IU/ml), 1-mM sodium pyruvate, non-essential amino acids are added to Roswell Park Memorial Institute medium 1640 (RPMI 1640). (Invitrogen, USA). Lonza, BioWhittaker, USA provided the ammonium-chloride-potassium (ACK) lysis buffer.

### Sample collection and maintenance

Fresh specimens *Padina pavonia, Taonia atomaria, Jania rubens *and *Ellisolandia elongata *(formerly *Corallina elongata*) were collected from Rocky Bay of Abu Qir, Alexandria, Egypt. Seaweeds were washed in seawater, then in tap water. The seaweeds were next shaded and air-dried at room temperature then at 38 ± 2 °C. The dried samples were broken-down to a fine powder and kept for further investigations. The seaweeds were identified in accordance with Jha et al. [30], and Kanaan and Belous [31] and the Algae base website [32].

### Preparation of seaweed extracts

The extraction was performed according to El-Sheekh et al. [33]. Briefly, one gram of seaweeds was steeped in methanol (1:30 w/v) in a conical flask for two days at room temperature on a rotary shaker at 120 rpm. The filtrate was placed in a 45 °C oven to remove the excess solvent. The crude extracts were suspended in the methanol to a final concentration of 5 mg/mL and stored at -20 °C for further studies. The extraction yield percentage was calculated according to Maisuthisakul and Pongsawatmanit [34]

$${\text{Extraction yield}}\% = \, \left( {{\text{W}}_{{1}} /{\text{W}}_{{2}} } \right) \times {1}00.$$

where W_1_ is the weight of dried crude extract, and W2 is the weight of the sample before extraction.

### Cell culture

Human liver cancer Hep-G2 cells lines (ATCC, Rockville, MD, USA) were cultured on complete RPMI-1640 medium supplied with heat-inactivated fetal bovine serum (FBS) (10% v/v), 2-mM l-glutamine, Penicillin/Streptomycin solution (100 IU/ml), 1-mM sodium pyruvate and non-essential amino acids. The cells were sub-cultured two to three times per week in a humid incubator (Sanyo XD-101; Sanyo) with 5% CO_2_ at 37 °C.

### In vitro *antitumor activity assay*

Hep-G2 cell lines were suspended in PBS buffer, and then they were added to the medium at a concentration of 5 × 10^4^ cell/well in 96-multi well plates. The plates were then incubated for 24 h. Following that, the tested substances were put onto 96-well plates at concentrations ranging from 10 to 1000 μg/mL, along with the reference drug Cisplatin^®^ at concentrations ranging from 5 to 100 µg/mL (three replicates). For each 96 well plate, three controls with media or 0.5% DMSO were conducted. After incubating for 24 and 48 h, the viable cells numbers were investigated by MTT assay. In brief, 96-well plate's media were removed, and replaced with 100 µL of fresh RPMI 1640 medium. Then, 10 µl of the 12 mM MTT (Sigma, USA) stock solution (5 mg of MTT/1 mL PBS) was added to each well. After that, the 96-well plates were incubated for 4 h at 37 °C with 5% CO_2_. 50 µL of DMSO was added to each well, thoroughly mixed with the pipette, and then incubated at 37 °C for 10 min. The optical density was determined at 590 nm with the microplate reader (Bio-Rad microplate reader, Japan).

The percentage of viability was calculated as:

$$\left( {\text{ODt/ODc}} \right) \, \times {1}00\% ,$$

where ODt is the mean optical density of wells treated with the tested sample and ODc is the mean optical density of untreated cells.

The survival curve of the tumor cell line after treatment with seaweed extracts is depicted using the relationship between surviving cells and drug concentration. The Graphpad Prism software (San Diego, CA, USA) was used to calculate the 50% inhibitory concentration (IC_50_) using graphic plots of the dose–response curve for each conc. [35]. The IC_50_ values for the HepG-2 cell line after 24 h were estimated at 613 µg/ml and 475.1 µg/ml for *P.* *pavonica* and *J.* *rubens*, respectively.

In vivo 50% lethal dose (LD_50_) values were calculated from the IC_50_ values according to the formula: log (LD_50_) = 0.372 × log (IC_50_) + 2.024 [36]*.* LD50 values were estimated at 1150.5 μg/kg and 1047.0 μg/kg for *P.* *pavonica* and *J.* *rubens*, respectively.

### Mice

Ninety female Swiss albino mice CD1 (6–8 weeks old, weighing 25 ± 2 g), were provided by Theodor Bilharz Research Institute (TBRI), Cairo, Egypt. Mice divided into nine groups (n = 10). Mice were given a standard pellet diet and tap water ad libitum. The experimentation was conducted in accordance with the National Institutes of Health guide for the care and use of laboratory animals, as well as the recommendations for the proper care and use of laboratory animals, regulated by the Faculty of Science, Tanta University, Tanta, Egypt.

### Tumor cell line and tumor model preparation

Ehrlich ascites carcinoma EAC cell line (National Cancer Institute, Cairo University, Cairo, Egypt) was kept in ascetic form in female Swiss albino mice by weekly intraperitoneal (i.p.) inoculation of 2.5 × 10^6^ cells/mouse [37]. The ascetic fluid was collected and diluted with PBS. EAC cells were count using Trypan Blue dye exclusion method. EAC cells were resuspended in PBS at a density of 5 × 10^6^ cells/mL PBS and kept at − 80 °C. To prepare the tumor model, 0.25 × 10^6^ EAC cells were i.p. implanted into naïve female Swiss albino mice.

### *Tumor challenge and* in vivo *study design*

**Fig. 1:**
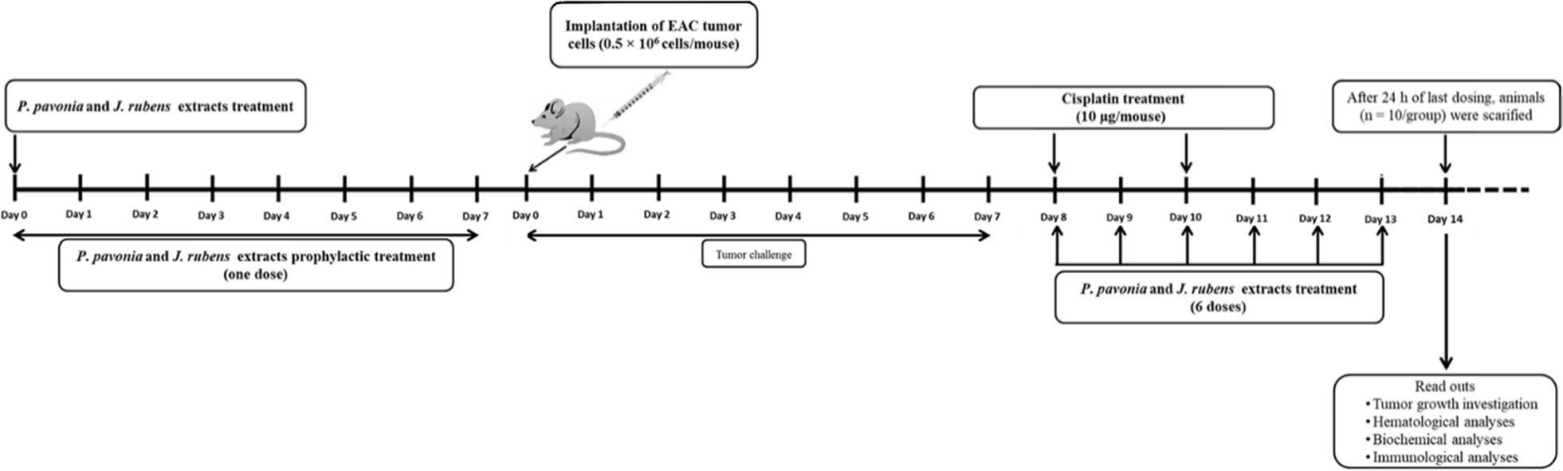
In vivo experiment dosage plan

Eighty female Swiss albino mice were divided into eight groups of 10 mice each. Groups 1–8 i.p transplanted with EAC cell line (0.25 × 10^6^ EAC cells/mouse). One day post EAC transplantation, group 1 was given i.p saline (naïve EAC control). Group 2 was i.p injected with standard drug Cisplatin^®^ (10 µg/mouse) (positive EAC control) on days 8 and 10 post EAC transplantation. on days 8, 9, 10, 11, 12, and 13 post EAC transplantation, group 3 and group 4 were i.p. inoculated with *P*. *pavonica* extract (2.5 μg/mouse, ten times less than LD_50_ and 1.3 μg/mouse, twenty times less than LD_50_, respectively), and Group 5 and group 6 were i.p injected with *J.* *rubens* extract (2.3 μg/mouse, ten times less than LD_50_ and 1.2 μg/mouse, twenty times less than LD_50_, respectively). Seven days before EAC transplantations, Group 7 and group 8 (prophylactic groups) were i.p inoculated with *P.* *pavonica* and *J.* *rubens* extracts (2.5, μg/mouse and 2.3 μg/mouse, ten times less than LD_50_, respectively). On day 14 post EAC transplantation, all groups of mice were sacrificed, immunological and antitumor efficacy were assessed, and sera were collected for hematological and biochemical analysis (Fig. 1).

### Hematological analysis

By the end of the experimental study, mice were anaesthetized with mild ether, and blood was drained from their retro-orbital plexus using heparinized microhematocrit tubes. A Nihon Kohden automated hematology analyzer (model MEK-6318K, Japan) was used to assess blood parameters, including leucocytes (WBC) (10^3^/cmm) and their differential relative percent (neutrophils, lymphocytes, basophils, and monocytes) in peripheral blood (PB).

### Preparation of sera

Blood was drawn from the retro-orbital plexus of experimental mice before they were euthanized by cervical dislocation. Blood samples were gathered in test tubes and left to stand for 3 h to ensure complete coagulation. After centrifugation at 3000 rpm for 10 min, the sera samples were sucked out and stored at − 80 °C for biochemical analysis.

### Tumor cells harvesting and counting

Mice were sacrificed by cervical dislocation, and immediately EAC ascetic were individually withdrawn from treated-EAC and nontreated-EAC mice using a syringe containing 5 mL of PBS. The cells were resuspended in PBS (Sigma Chemical Co., St. Louis, USA) and washed twice. Red blood cells were lysed with ammonium chloride potassium buffer (ACK; Invitrogen, Carlsbad, CA, USA). EAC cells suspensions were centrifuged for 5 min (3000 rpm) at 4 °C, discarding the supernatant. The EAC cell pellets were washed and diluted in supplemented PBS. EAC cells were counted using a Neubauer hemocytometer, and the cell viability was determined using a trypan blue dye exclusion assay. Viability was calculated by trypan blue exclusion assay and consistently exceeded 90%.

### Flow cytometry

Fresh single-cell suspensions of tumor EAC cells were collected from the ascites of testing mice. 1 × 10^6^ cells were stained with anti-mouse CD4, anti-mouse CD8, and anti-mouse CD335 (NKp46), which were then incubated for 30 min at room temperature in the dark before being cooled on ice for 1 min. The cells were washed twice and resuspended in 0.3 mL of 0.5% bovine serum albumin and 0.02% sodium azide solution. Flow cytometry was used to detect the CD4 + T, CD8 + T, and CD335 cell subsets using a FACS Calibur system (BD Biosciences, San Jose, CA); all data analysis was done with Cell-Quest software (Becton Dickinson, San Jose, CA, USA). For each sample, a minimum of 50,000 events was acquired. In each compartment, the total numbers of different cell populations were calculated each time.

### Assessment of apoptosis by flow cytometry

Treated EAC cells from Cisplatin^®^-treated EAC mice, seaweed extracts-treated EAC mice, and nontreated EAC mice were stained with FITC Annexin V (Apoptosis Detection Kit II; Cat. No 556570 BD Bioscience, USA) following the manufacturer's instructions. The data was evaluated using a BD FACSCanto II flow cytometer (BD Becton, Dickinson Company, USA) and analyzed using BD FACS Diva software (BD Becton, Dickinson Company, USA) (BD Bioscience, USA). The apoptosis by Annexin V FITC/PI Assay for EAC tumor cells collected from the peritoneal cavity of tumor-bearing mice after treatment was gated on live cells on FSC v SSC, then single cells were gated using the PI width (W) and area (A) dot plot. Staining by Annexin V is used in association with a vital dye propidium iodide (PI) to recognize early and late apoptotic cells. Viable cells with intact membranes keep PI out, whereas dead and damaged cells' membranes let it in. As a result, cells are considered viable when they are both Annexin V and PI negative. Cells in early apoptosis, on the other hand, are Annexin V positive and PI negative. Annexin V and PI are both positive in late apoptosis and dead cells.

### Analysis of liver and kidney functions

The levels of serum aspartate aminotransferase (AST) (U/l), alanine aminotransferase (ALT) (U/l), albumin (g/dL), creatinine (mg/dL) were determined colorimetrically using standard ready-to-use kits and methods of Human (Human Gesellschaft Für Biochemica and Diagnostica MBH, Germany) by a fully automated biochemistry analyzer (Vita lab Selectra E, German). Throughout the experiment, the manufacturer's instructions for each biochemical parameter were strictly followed.

### Statistical analysis

The numerical data was reported as a mean ± SD. Statistical analyses was carried out by a one-way analysis of variance (ANOVA). Statistical significance was determined by a Tukey and a post-hoc test followed by Dunnett’s multiple comparison tests. Statistical comparisons between groups were done using the paired Student’s t-test. Statistical. Significant differences were considered at *p*-values < 0.0 5.

## Results

### In vitro *antitumor activity*

In the present study, the methanol extract of four algal species: *Jania* *rubens,* *Padina* *pavonica,* *Taonia* *atomaria* (Phaeophyceae)*,* and *Ellisolandia* *elongata* (formerly *Corallina* *elongata*) (Rhodophyta) with different concentrations (10–1000 μg/mL) were tested for its growth inhibition activity against human liver cancer HepG2 cell line (24 h incubation) with MTT assay. The human liver cancer HepG2 assayed with methanolic extracts of *J.* *rubens,* *T.* *atomaria*, *P.* *pavonica,* and *E.* *elongata* resulted in *J.* *rubens* extract has imparted 10.6 ± 3.7 to 77.0 ± 0.95% growth inhibition with a decrease in concentration and IC_50_ of 475.1 μg/mL, *T.* *atomaria* extract has imparted 16.1 ± 2.8 to 68.3 ± 2.3% growth inhibition with a decrease in concentration and IC_50_ of 580 μg/mL, *P.* *pavonica* extract has imparted 19.7 ± 1.3 to 61.6 ± 9.3% growth inhibition with a decrease in concentration and IC_50_ of 613 μg/mL, and *E.* *elongata* extract has imparted 22.0 ± 5.8 to 56.5 ± 1.5% growth inhibition with a decrease in concentration and IC_50_ of 630.9 μg/mL (Table1). It is observed a crude extract of *J.* *rubens* is influencing the increase in cell growth inhibition in the human liver cancer HepG2 when compared to that of *T.* *atomaria,* *P.* *pavonica* and *E.* *elongata* *with* IC_50_ values at 475.1 μg/ml, 580 μg/ml, 613. μg/mL, and 630.9 μg/mL for *J.* *rubens,* *T.* *atomaria,* *P.* *pavonica,* and *E.* *elongata,* respectively (Table 1). Depending on the trypan blue assay, *P.* *pavonica* *and* *J.* *rubens* methanol extracts were selected for advanced studies to determine immunomodulatory and antitumor potentials.

**Table 1:** In vitro cytotoxic effect of *P.* *pavonica,* *J.* *rubens*, *T.* *atomaria* and *E.* *elongata* extract by MTT assay at various concentration (10–1000 µg/ml) against human liver cancer Hep-G2 cell line at 24 h-incubation period

Extract concentration (µg/ml)	% Growth inhibition			
	*P.* *pavonica*	*J.* *rubens*	*T.* *atomaria*	*E.* *elongata*
10	19.70 ± 1.30^a^	10.60 ± 3.70	16.10 ± 2.80a	22.00 ± 5.80a
50	38.00 ± 3.20^a^	14.30 ± 2.10	21.90 ± 3.90a	27.40 ± 1.80a
100	40.50 ± 5.10^a^	23.20 ± 3.90a	21.40 ± 7.60a	29.40 ± 3.40a
200	39.30 ± 0.80^a^	27.40 ± 3.20a	40.50 ± 10.70a	49.90 ± 14.80a
400	42.30 ± 1.10^a^	66.40 ± 2.00a	46.00 ± 7.20a	48.30 ± 2.40a
600	51.20 ± 11.70^a^	66.80 ± 2.80a	54.10 ± 1.10a	51.20 ± 6.200^a^
800	54.10 ± 2.10^a^	68.50 ± 2.80a	57.90 ± 3.60a	55.10 ± 2.60^a^
1000	61.60 ± 9.30^a^	77.00 ± 0.95a	68.30 ± 2.30a	56.50 ± 1.50^a^
Control	00.00 ± 0.00	0.000 ± 0.00	00.00 ± 0.00	00.00 ± 0.00
IC_50_ value (µg/mL)	613.00	475.10	580.00	630.90

Data were represented as mean ± SD

The difference between groups was considered statistically significant at *P* < 0.05. *Statistically significant vs. control at 24 h-incubation period.

Our result showed that in vitro treatment of human liver cancer Hep-G2 with reference therapeutic drug Cisplatin^®^ with different concentrations (5–100 μg/ml) resulted in impairing of 20.90 ± 1.50 to 49.50 ± 6.70% growth inhibition with a decrease in concentration at IC_50_ of 90.40 μg/ml (Table 2).

**Table 2:** In vitro cytotoxic effect of standard chemotherapeutic drug Cisplatin^®^ by MTT assay at various concentration (5–100 µg/mL) against human liver cancer Hep-G2 cell line at 24 h-incubation period

Concentration (µg/mL)	% Growth inhibition
5	20.90 ± 1.50^a^
10	35.20 ± 1.70^a^
20	39.60 ± 3.90^a^
40	41.80 ± 5.00^a^
80	48.30 ± 5.90^a^
100	49.50 ± 6.70^a^
Control	00.00 ± 0.00
IC_50_ value (µg/mL)	90.40

Data were represented as mean ± SD

The difference between groups was considered statistically significant at *P* < 0.05. *Statistically significant vs. control at 24 h-incubation period

In vivo 50% lethal dose (LD_50_) values were calculated from the IC_50_ values according to the formula: log (LD_50_) = 0.372 × log (IC_50_) + 2.024*.* LD50 values were estimated at 1150.5 μg/kg and 1047.0 μg/kg for *P.* *pavonica* and *J.* *rubens*, respectively. Two sublethal doses of *P*. *pavonica* and *J.* *rubens* has been chosen for continued immunological and antitumor assays: 2.5 μg/mouse (ten times less than LD_50_) and 1.3 μg/mouse (twenty times less than LD_50_) for *P*. *pavonica* extract and 2.3 μg/mouse (ten times less than LD_50_) and 1.2 μg/mouse (twenty times less than LD_50_) for *J.* *rubens* extract.

### Changes in the level of leucocytes profile

Treatment of EAC mice with a low dose of *P.* *pavonica* extract (1.3 μg/mouse) and a prophylactic dose of *J.* *rubens* extract (2.3 μg/mouse) significantly increased the total number of WBCs by about 2.2 and 2.5 folds, respectively compared to naive EAC mice and by 1.9 and 2 folds, respectively compared to Cisplatin^®^-treated EAC mice. Furthermore, treatment with a high dose of *J.* *rubens* extract (2.3 μg/mouse) induced a significant elevation in WBCs total number by 2 and 1.5 folds compared to naive EAC mice and Cisplatin^®^-treated EAC mice, respectively. Surprisingly, treatment with a high dose of *P.* *pavonica* extract (2.5 μg/mouse) and a low dose of *J.* *rubens* extract (1.2 μg/mouse) exhibited a significant decrease in total WBCs by about 0.5 fold compared to naïve EAC mice (Table 3).

**Table 3:** Cellular alternation in peripheral blood leucocytes profile of EAC mice treated with Cisplatin^®^, *p.* *pavonica* and *J.* *ruben* *extracts*

Treatments		WBC count (× 0^3^)	Leucocytes differentials relative number (%)		
			Lymphocytes	Neutrophils	Monocytes
*P.* *pavonica*	Low dose	11.96 ± 0.60^a^	77.00 ± 7.93	4.66 ± 0.08	24.33 ± 3.05
	High dose	2.35 ± 0.50	76.00 ± 3.60	2.00 ± 0.05^a^	21.33 ± 2.52
	Prophylactic dose	5.36 ± 0.66	76.33 ± 6.42	4.33 ± 0.09	18.66 ± 2.23
*J.* *rubens*	Low dose	6.46 ± 0.30	86.00 ± 5.30	1.66 ± 0.04^a^	12.00 ± 3.29
	High dose	3.63 ± 0.93	78.00 ± 6.00	4.00 ± 0.09^a^	17.33 ± 4.32
	Prophylactic dose	9.23 ± 1.40^ab^	76.66 ± 8.50	1.66 ± 0.02^a^	21.66 ± 1.02
EAC + PBS		5.20 ± 0.70	72.66 ± 9.72	7.66 ± 0.06	23.66 ± 3.06
EAC + Cisplatin^®^		6.46 ± 0.50	70.00 ± 7.64	1.33 ± 0.08	25.33 ± 2.50

Data were represented as mean ± SD

The difference between groups was considered statistically significant at *P* < 0.05. ^a^: statistically significant vs. EAC mice. ^b^: statistically significant vs. EAC mic + Cisplatin®

Treatment of EAC mice with a low dose, a high, and prophylactic dose of *P.* *pavonica* extracts (1.3 μg/mouse, 2.5 μg/mouse, and 2.5 μg/mouse, respectively) and that of *J.* *rubens* extracts (1.2 μg/mouse, 2.3 μg/mouse and 2.3 μg/mouse, respectively) resulted in the remarkable decreases in neutrophil relative% compared to naïve EAC mice. Noticeably, treatment of EAC mice at a high dose of *P.* *pavonica* extract (2.5 μg/mouse) and low dose and prophylactic dose (2.3 μg/mouse) of *J.* *rubens* extract significantly decreased the neutrophil relative% compared to naïve EAC mice. Remarkably, *P.* *pavonica* extract at low and prophylactic doses and *J.* *rubens* at high dose increased the neutrophil relative% compared to Cisplatin^®^-treated EAC mice (Table 3).

Intraperitoneal inoculation of EAC mice with *P.* *pavonica* and *J.* *rubens* extracts at low, high and prophylactic doses resulted in a nonsignificant increase of lymphocytes relative% compared to naïve EAC and Cisplatin^®^-treated EAC mice. Furthermore, among all tested doses, a low dose of *J.* *rubens* recorded the highest lymphocytes percentage compared to Naïve EAC and Cisplatin^®^-treated EAC mice (Table 3).

Intraperitoneal treatment of EAC mice with *J.* *rubens* extracts at low, high and prophylactic doses led to a remarkable nonsignificant decrease of monocytes relative% reached the least value at low dose compared to naïve EAC and Cisplatin^®^-treated EAC mice. Treatment of EAC mice with *P.* *pavonica* extracts at high and prophylactic doses led to a nonsignificant decrease of monocytes relative% compared to naïve EAC and Cisplatin^®^-treated EAC mice (Table 3).

### *Antitumor effects of different doses of seaweeds extract on EAC growth* in vivo

**Fig. 2:**
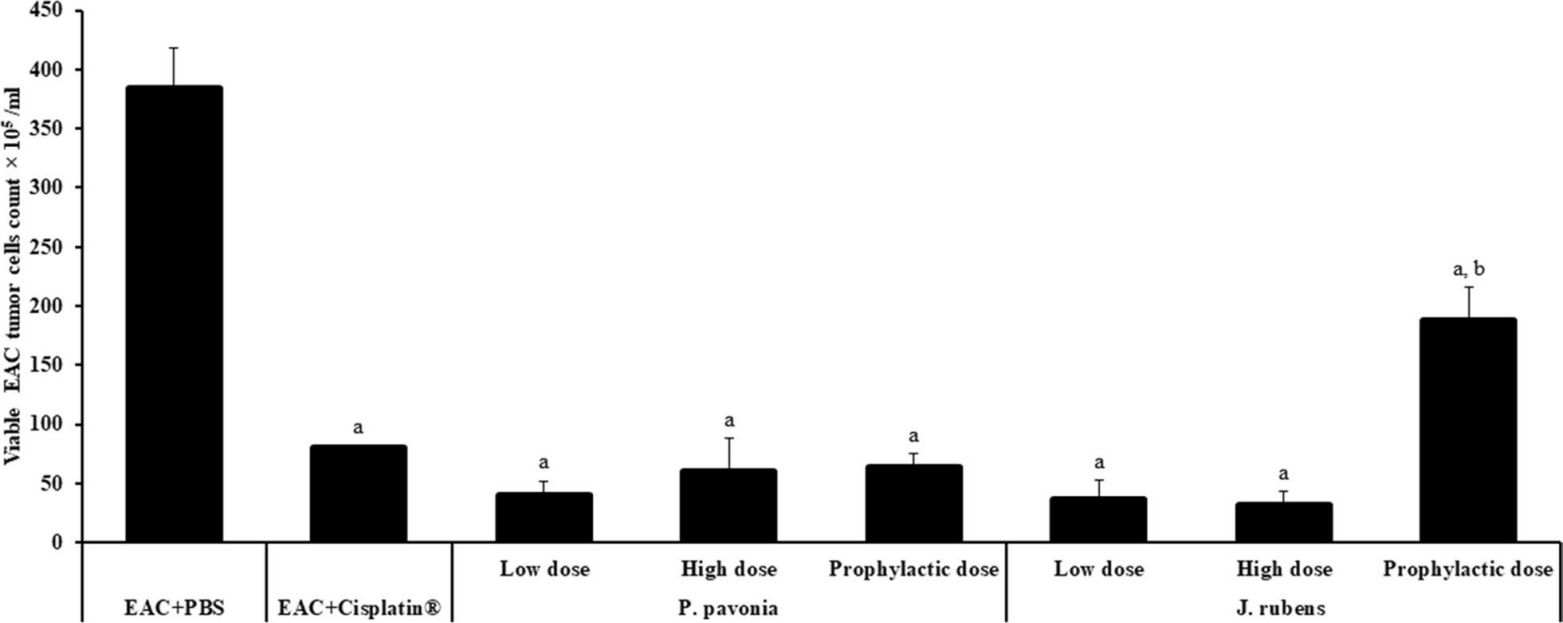
Antitumor effects of *p.* *pavonica* and *J.* *ruben* extracts on EAC tumor growth in vivo*:* Mice (n = 10) were i.p inoculated with *p.* *pavonica* at low dose (1.3 μg/mouse), high dose (2.5 μg/mouse) or prophylactic dose (2.5 μg/mouse), *J.* *rubens* at low dose (1.2 μg/mouse), high dose (2.3 μg/mouse) or prophylactic dose (2.3 μg/mouse), Cisplatin^®^ (10 μg/mouse) or phosphate buffer saline (PBS). Data were represented as mean ± SD. The difference between groups was considered statistically significant at *P* < 0.05. ^a^Statistically significant vs. EAC mice. ^b^Statistically significant vs. EAC mic + Cisplatin^®^

Interestingly, high, low, and prophylactic doses of *P.* *pavonica* extract (1.3 μg/mouse, 2.5 μg/mouse, and 2.5 μg/mouse, respectively) and *J.* *rubens* extracts at low, high and prophylactic doses of (1.2 μg/mouse and 2.3 μg/mouse, 2.3 μg/mouse, respectively) produced a significant decrease in the number of viable EAC harvested tumor cells compared to naïve EAC mice (Fig. 2). Importantly our findings revealed that i.p injection of EAC mice with *P.* *pavonica* extract at low, high and prophylactic doses (1.3 μg/mouse, 2.5 μg/mouse, and 2.5 μg/mouse, respectively) and *J.* *rubens* extracts at low and high doses of (1.2 μg/mouse and 2.3 μg/mouse, respectively) remarkably decreased the total count of EAC tumor cells compared to Cisplatin^®^-treated EAC mice. However, these effects were higher in EAC mice i.p inoculated with a prophylactic dose of *J.* *rubens* extract compared to the effect reported in EAC mice i.p treated with Cisplatin^®^ (Fig. 2).

### Flow cytometric analysis of CD4, CD8, and CD335

**Fig. 3:**
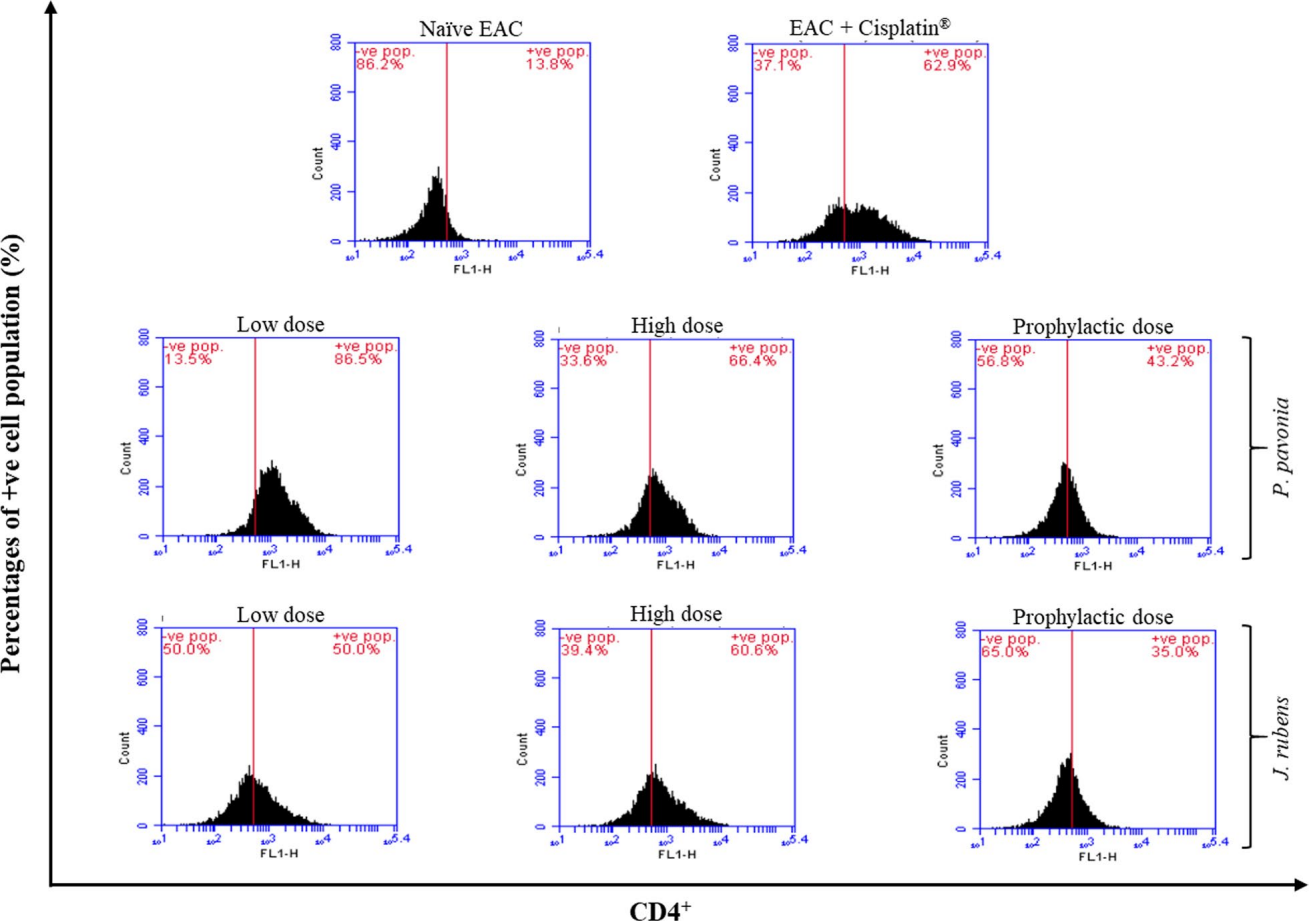
Flow cytometric analysis of Phenotypic modifications helper CD4^+^ T cells induced in EAC mice by treatment with *P.* *pavonica* and *J.* *rubens* extracts: Mice (n = 10) were i.p inoculated with *P.* *pavonica* at low dose (1.3 μg/mouse), high dose (2.5 μg/mouse) or prophylactic dose (2.5 μg/mouse), *J.* *rubens* at low dose (1.2 μg/mouse), high dose (2.3 μg/mouse) or prophylactic dose (2.3 μg/mouse), Cisplatin^®^ (10 μg/mouse) or phosphate buffer saline (PBS)

**Fig. 4:**
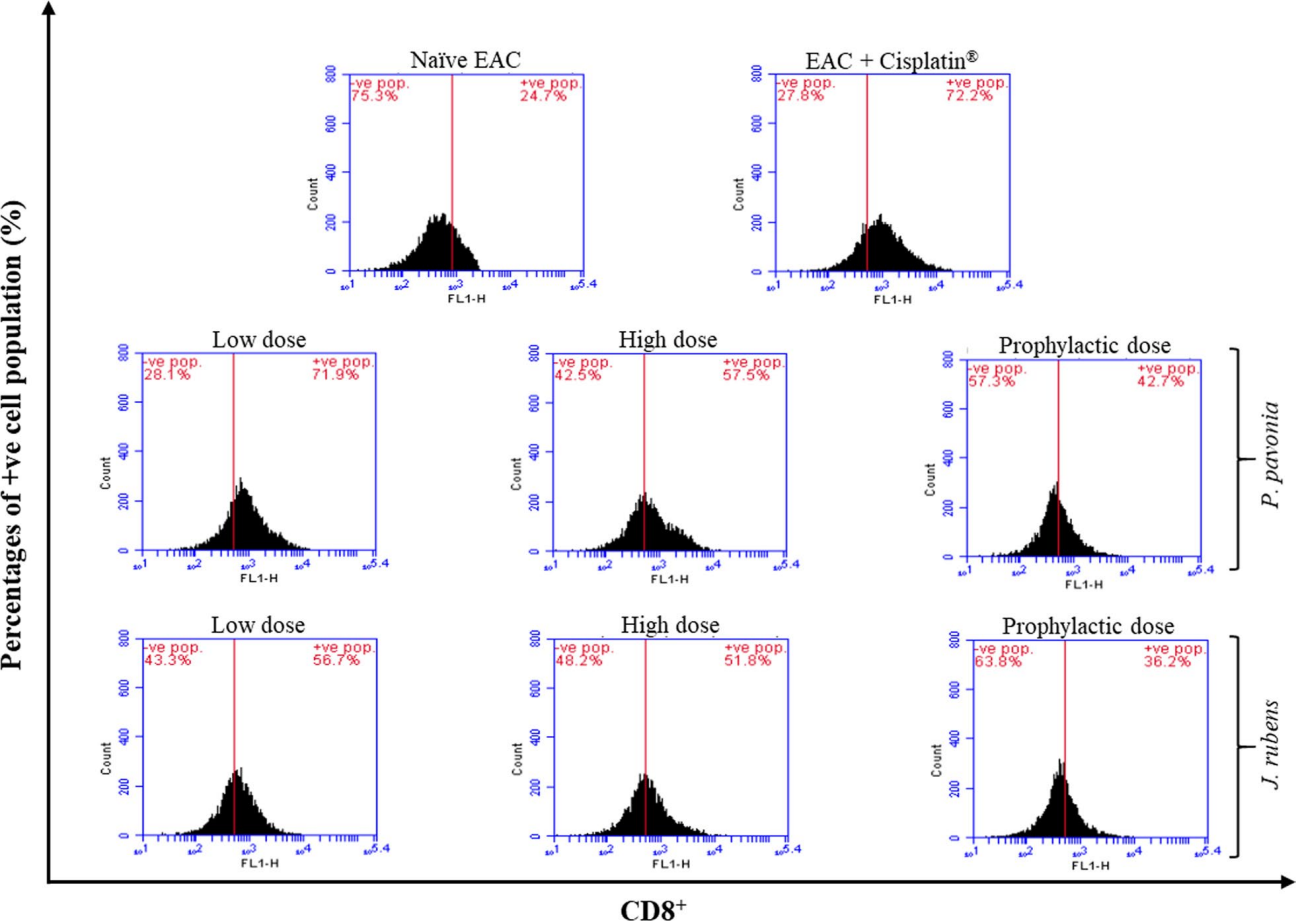
Flow cytometric analysis of phenotypic modifications cytotoxic CD8^+^ T cells induced in EAC mice by treatment with *p.* *pavonica* and *J.* *rubens* extracts: Mice (n = 10) were i.p inoculated with *p.* *pavonica* at low dose (1.3 μg/mouse), high dose (2.5 μg/mouse) or prophylactic dose (2.5 μg/mouse), *J.* *rubens* at low dose (1.2 μg/mouse), high dose (2.3 μg/mouse) or prophylactic dose (2.3 μg/mouse), Cisplatin^®^ (10 μg/mouse) or phosphate buffer saline (PBS)

**Fig. 5:**
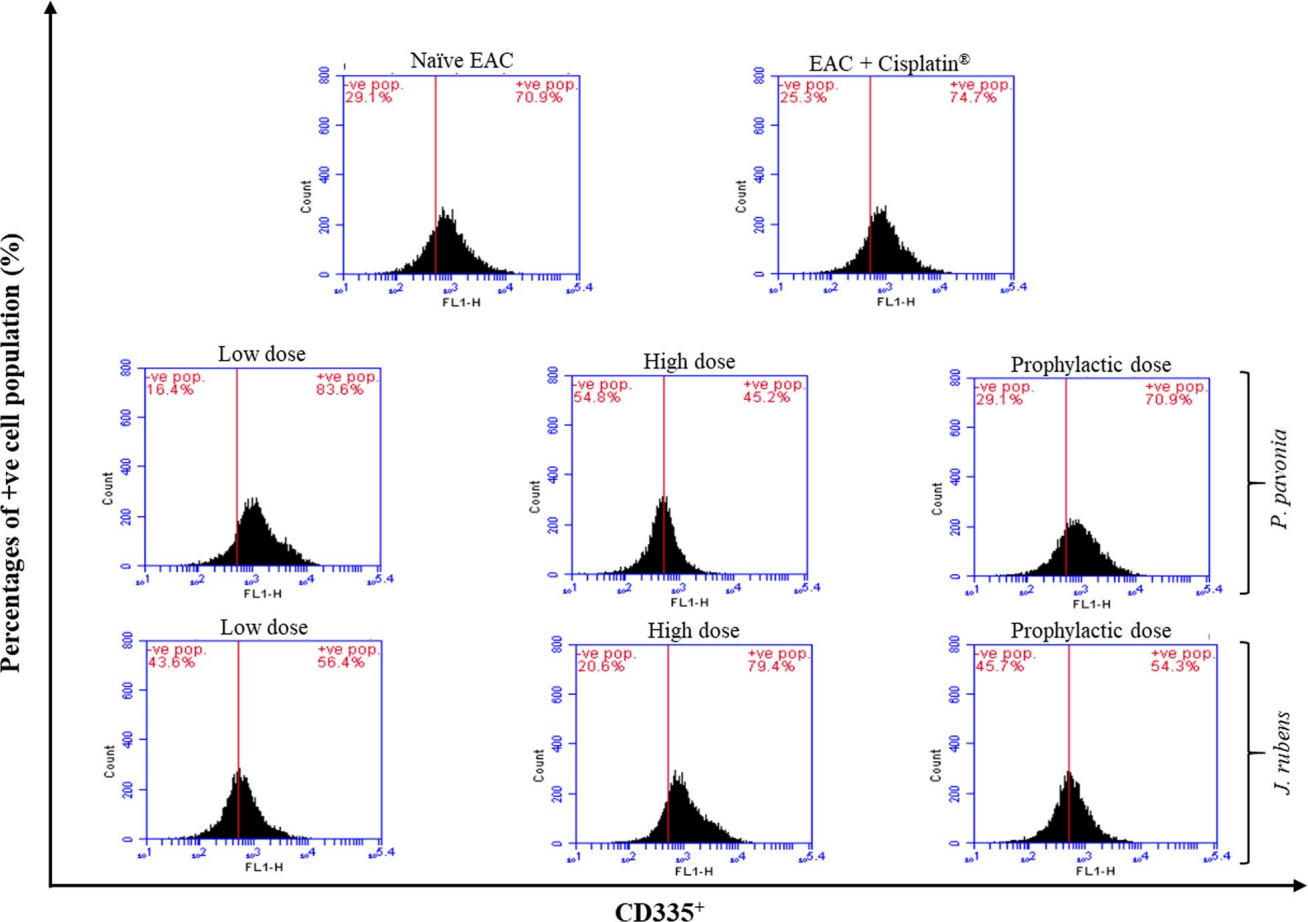
Flow cytometric analysis of Phenotypic modifications natural killer (NK) CD335^+^ cells induced in EAC mice by treatment with *p.* *pavonica* and *J.* *rubens* extracts: Mice (n = 10) were i.p inoculated with *p.* *pavonica* at low dose (1.3 μg/mouse), high dose (2.5 μg/mouse) or prophylactic dose (2.5 μg/mouse), *J.* *rubens* at low dose (1.2 μg/mouse), high dose (2.3 μg/mouse) or prophylactic dose (2.3 μg/mouse), Cisplatin^®^ (10 μg/mouse) or phosphate buffer saline (PBS)

The possible proliferative and inhibition efficacy of *P.* *pavonica* and *J.* *rubens* extracts on the phenotypic expressions of splenic. lymphocytes subsets (helper CD4^+^ T cells, cytotoxic CD4^+^ T cells, and natural killer NK CD335 cells) have been investigated with anti-mouse, anti-mouse CD_4_, CD_8,_ and anti-mouse CD335 staining using flow cytometry (Figs. 3, 4 and 5). Intraperitoneal treatment of EAC mice groups with *P.* *pavonica* extract at low, high and prophylactic doses (1.3 μg/mouse, 2.5 μg/mouse, and 2.5 μg/mouse, respectively) and *J.* *rubens* extract at low, high and prophylactic doses of (1.2 μg/mouse and 2.3 μg/mouse, 2.3 μg/mouse, respectively) increased the percentage of splenic helper CD4^+^ T cells by 6.3, 4.8 and 3.1-fold for *P.* *pavonica* extract and 3.6, 4.4 fold and 2.5 for *J.* *rubens* extract, respectively compared to naïve tumor mice (Fig. 3). Interestingly, i.p. inoculation of EAC mice with a low dose of *P.* *pavonica* extract and high dose of *J.* *rubens* extracts recorded the highest increase in the percentage of splenic helper CD4^+^ T cells subset population (86.5% and 60.6%, respectively) compared to naïve tumor mice (13.8%). The highest proliferative activity of helper CD4^+^ T cells was evaluated in the EAC mice i.p. inoculated with a low dose of *P.* *pavonia* extract (86.5%) compared to Cisplatin^®^-treated EAC mice (62.9%) (Fig. 3).

As shown in Fig. 4, phenotypic expressions of cytotoxic T cell subset of spleen harvested from EAC-bearing mice injected with *P.* *pavonica* and *J.* *rubens* extracts were detected with anti-mouse CD8 staining using flow cytometry. EAC mice i.p. received *P.* *pavonica* extract at low, high and prophylactic doses (1.3 μg/mouse, 2.5 μg/mouse, and 2.5 μg/mouse, respectively) and *J.* *rubens* extract at low, high and prophylactic doses of (1.2 μg/mouse and 2.3 μg/mouse, 2.3 μg/mouse, respectively)  showed a remarkable increase in the percentage of splenic cytotoxic CD8^+^ T cells by 2.9, 2.3 and 1.7 fold for *P.* *pavonica* extract and 2.3, 2.0 and 1.5 fold for *J.* *rubens* extract, respectively compared to naïve EAC mice (Fig. 4). Attractively, i.p. administration of EAC mice with a low dose of *P.* *pavonica* extract and *J.* *rubens* recorded the highest increase in the percentage of splenic cytotoxic CD8^+^ T cells subset population (71.9% and 56.7%, respectively) compared to naïve tumor mice (24.7%) (Fig. 4).

As shown in Fig. 5, phenotypic expressions of natural killer NK cell subset of spleen harvested from EAC mice injected with *P.* *pavonica* and *J.* *rubens* extracts were investigated with anti-mouse CD355(NKP46) staining by flow cytometry. EAC mice i.p. inoculated with *P.* *pavonica* extract at low doses (1.3 μg/mouse) and *J.* *rubens* extract at high dose (2.3 μg/mouse) showed a noticeable increase in the percentage of splenic natural killer NK cells by 1.2-fold for *P.* *pavonica* extract and 1.1 fold for *J.* *rubens* extract compared with naïve tumor mice (Fig. 5). Remarkably, i.p. treatment of EAC mice with the low dose of *P.* *pavonica* extract (1.3 μg/mouse) and the high dose of *J*. *rubens* extract (2.3 μg/mouse) expresses the highest increase in the percentage of splenic natural killer NK cell subset population (83.6% and 79.4%, respectively) compared to Cisplatin^®^-treated EAC mice (74.4%) (Fig. 5).

### Assessment of tumor apoptosis by flow cytometry

**Fig. 6:**
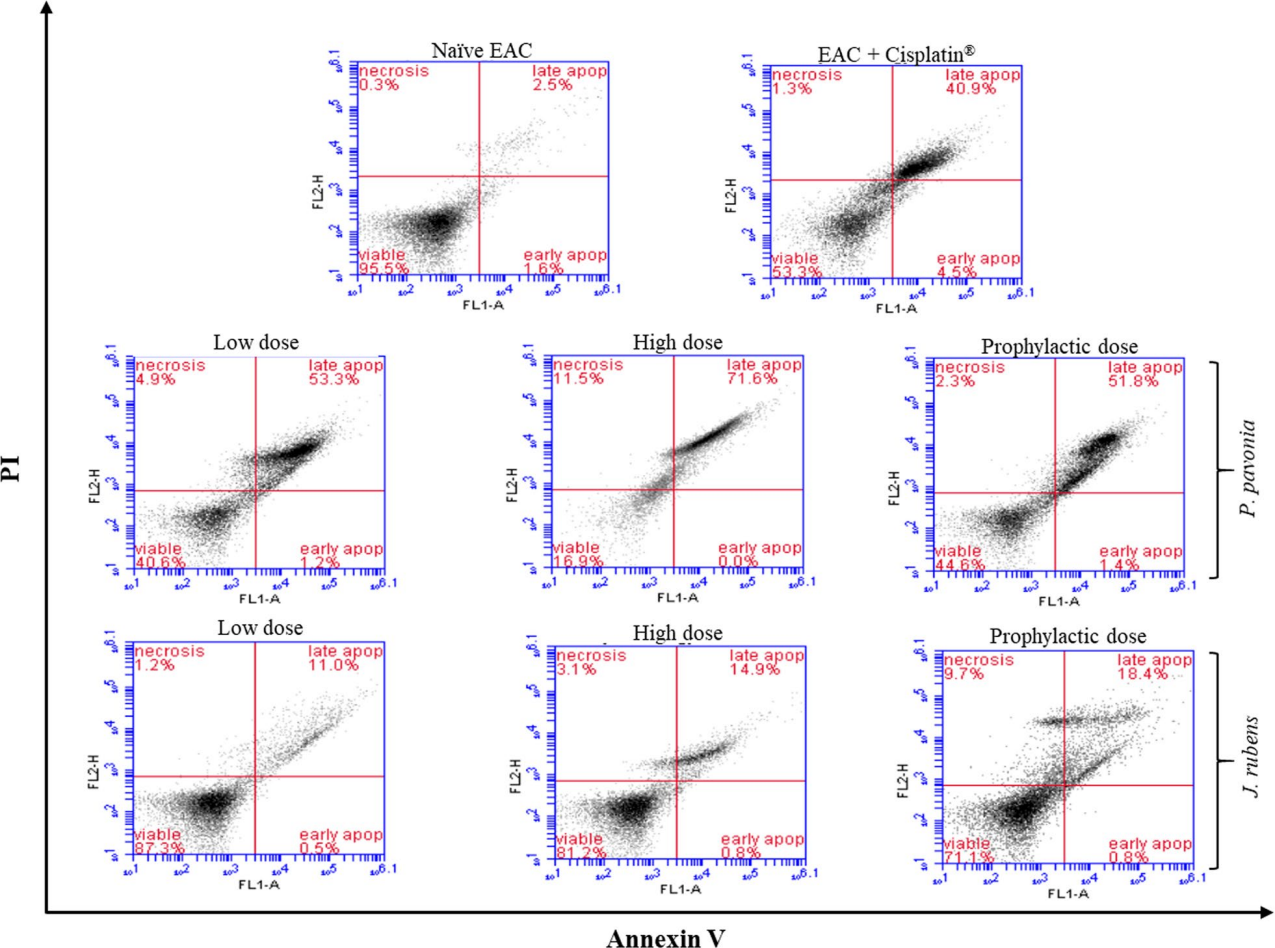
Flow cytometric analysis by Annexin-V/propidium iodide (PI) staining method of EAC tumor cells harvested from EAC mice treated with *P.* *pavonica* and *J.* *rubens* extracts: Mice (n = 10) were i.p. inoculated with *P.* *pavonica* at low dose (1.3 μg/mouse), high dose (2.5 μg/mouse) or prophylactic dose (2.5 μg/mouse), *J.* *rubens* at low dose (1.2 μg/mouse), high dose (2.3 μg/mouse) or prophylactic dose (2.3 μg/mouse), Cisplatin® (10 μg/mouse) or phosphate buffer saline (PBS)

In the present study, the antitumor efficacy of seaweeds extracts treatment regimen consisting of methanolic extracts of *P.* *pavonica* and *J.* *rubens* was assessed. EAC mice were i.p. injected with *P.* *pavonica* and *J.* *rubens* at low, high, and prophylactic doses and the apoptotic architecture of ascetic tumor cells were flowcytometrically investigated (Fig. 6). Our data reported that the i.p. treatment of EAC mice with *P.* *pavonica* extract at low, high and prophylactic doses (1.3 μg/mouse, 2.5 μg/mouse, and 2.5 μg/mouse, respectively) and *J.* *rubens* extract at low, high and prophylactic doses (1.2 μg/mouse, 2.3 μg/mouse and 2.3 μg/mouse, correspondingly) produced remarkable increases in the percentage of apoptosis of Ascetic EAC tumor cells at 53.3, 71.6, and 51.8%, respectively for *P.* *pavonica* extract and 11.0, 14.9 and 18.4%, respectively for *J.* *rubens* comparing to naïve tumor mice (2.5%) (Fig. 6). Interestingly, the treatment of EAC mice with *P.* *pavonica* extract at low, high and prophylactic doses (1.3 μg/mouse, 2.5 μg/mouse, and 2.5 μg/mouse, correspondingly) induced the highest apoptotic effect on EAC tumor cells by 1.3, 1.8 and 1.3-fold compared to a positive control (Cisplatin®-treated EAC mice) (Fig. 6). Meanwhile, treatment with *J.* *rubens* extract at low, high and prophylactic doses (1.2 μg/mouse, 2.3 μg/mouse, and 2.3 μg/mouse, correspondingly) managed to decrease the total count of EAC cells by 4.4, 5.96 and 7.4 folds, respectively compared to naïve tumor mice. However, these effects were much lower than the effect of cisplatin^®^ decreasing the number of harvested cells by 16.4 folds (Fig. 6).

### Liver and kidney function

**Fig. 7:**
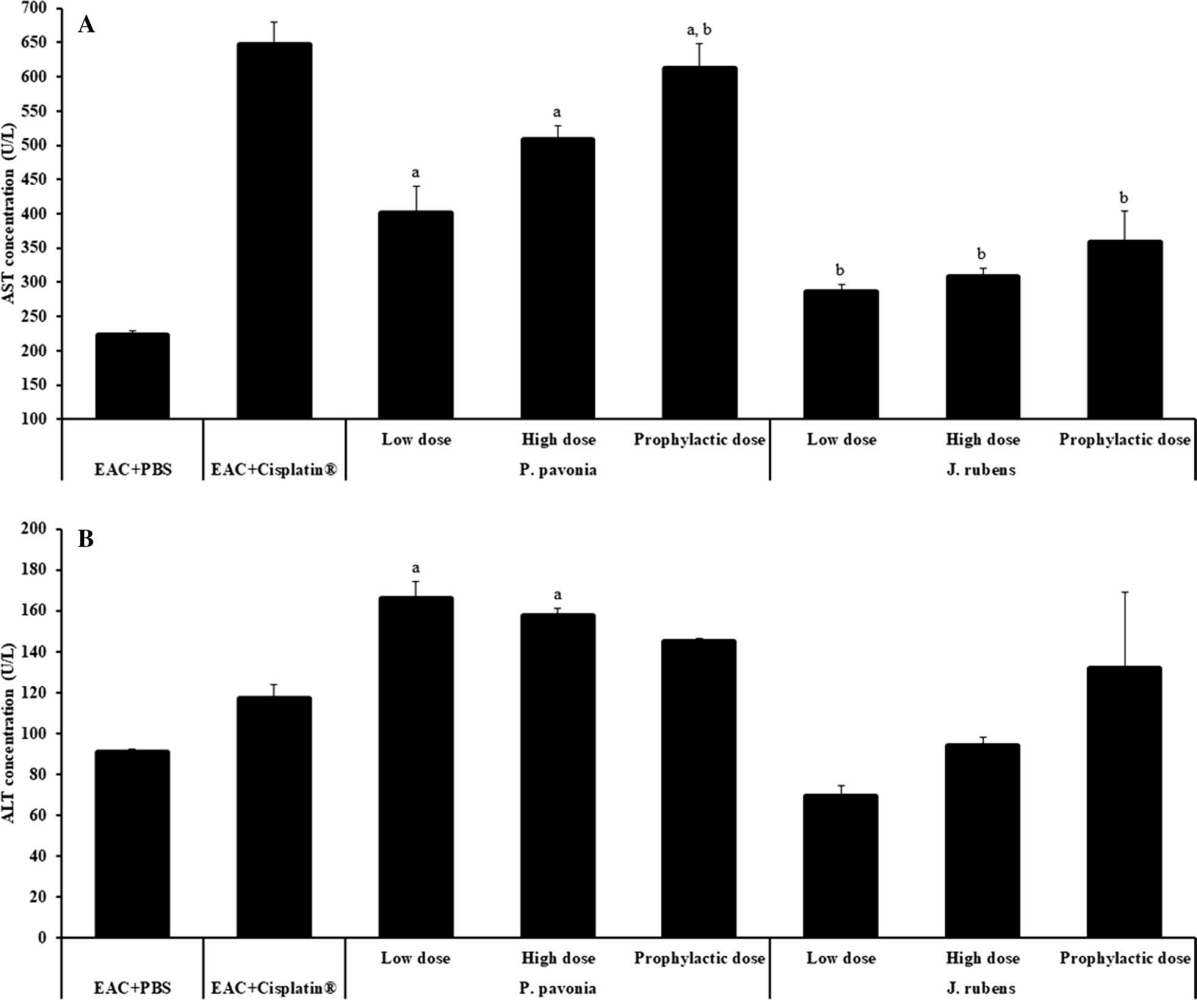
The effects *p.* *pavonica* and *J.* *rubens* extracts on liver function in EAC–bearing mice: Mice (n = 10) were i.p inoculated with *p.* *pavonica* at low dose (1.3 μg/mouse), high dose (2.5 μg/mouse) or prophylactic dose (2.5 μg/mouse), *J.* *rubens* at low dose (1.2 μg/mouse), high dose (2.3 μg/mouse) or prophylactic dose (2.3 μg/mouse), Cisplatin^®^ (10 μg/mouse) or phosphate buffer saline (PBS). Data were represented as mean ± SD. The difference between groups was considered statistically significant at *P* < 0.05. ^a^Statistically significant vs. EAC mice. ^b^Statistically significant vs. EAC mic + Cisplatin^®^

**Fig. 8:**
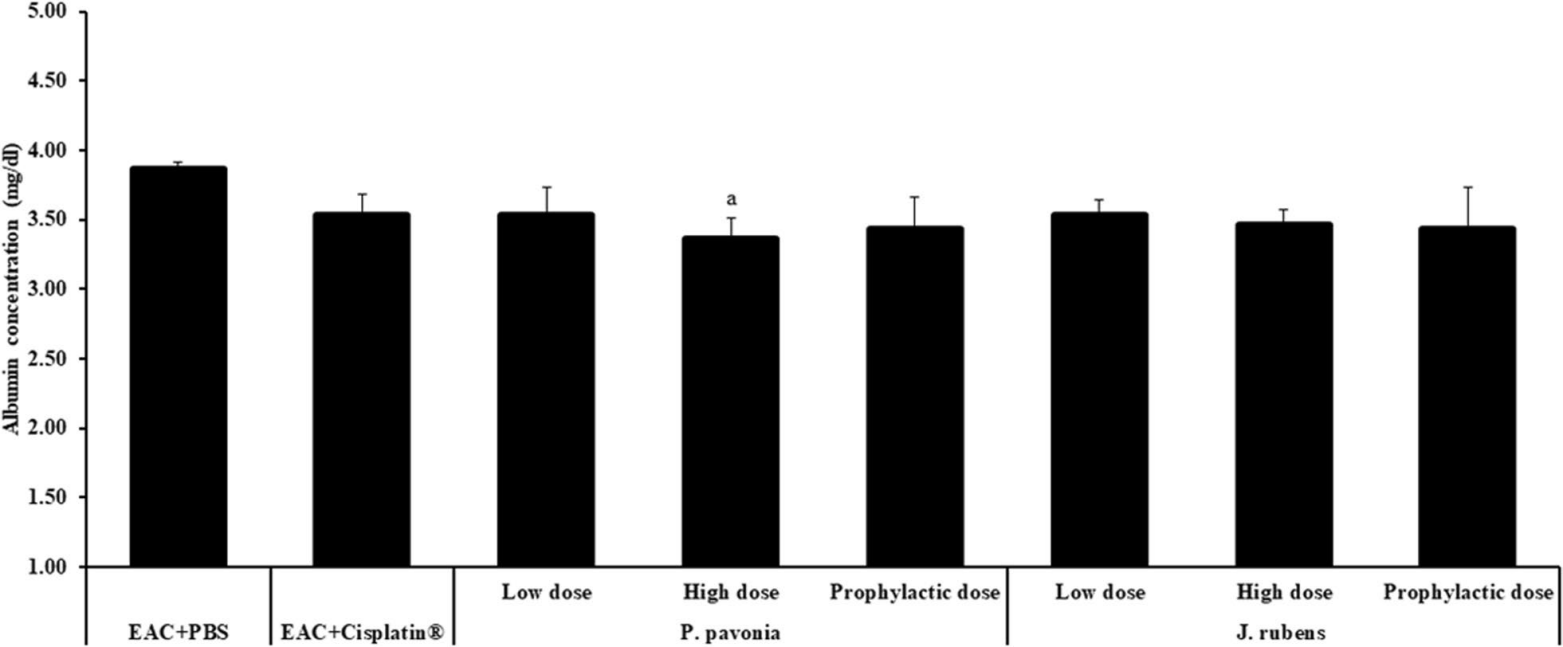
The effects *p.* *pavonica* and *J.* *rubens* extracts on Albumin concentration in EAC–bearing mice: Mice (n = 10) were i.p inoculated with *p.* *pavonica* at low dose (1.3 μg/mouse), high dose (2.5 μg/mouse) or prophylactic dose (2.5 μg/mouse), *J.* *rubens* at low dose (1.2 μg/mouse), high dose (2.3 μg/mouse) or prophylactic dose (2.3 μg/mouse), Cisplatin^®^ (10 μg/mouse) or phosphate buffer saline (PBS). Data were represented as mean ± SD. The difference between groups was considered statistically significant at *P* < 0.05. ^a^Statistically significant vs. EAC mice

Treatment with *J.* *rubens* extract at low, high and prophylactic doses (1.2 μg/mouse, 2.3 μg/mouse, and 2.3 μg/mouse, respectively) and *P.* *pavonica* extract at high and prophylactic doses (2.5 μg/mouse) had a significant decrease in AST level compared to Cisplatin®-treated EAC mice. Meanwhile, i.p. inoculation of *P.* *pavonica* extract at low, high, and prophylactic doses (1.3 μg/mouse, 2.5 μg/mouse, and 2.5 μg/mouse, respectively) had a significant raising in AST level compared to naïve EAC mice (Fig. 7). Intraperitoneal injection with *P.* *pavonica* extract at low, high and prophylactic doses (1.3 μg/mouse, 2.5 μg/mouse, and 2.5 μg/mouse, respectively) and *J.* *rubens* extract at prophylactic doses (2.5 μg/mouse) had a nonsignificant increase in ALT level compared to Cisplatin®-treated EAC mice. Meanwhile, i.p inoculation of *P.* *pavonica* extract at low and high doses (1.3 μg/mouse and 2.5 μg/mouse respectively) resulted in a significant increase in ALT level compared to naïve EAC mice (Fig. 7). Regarding the inoculation of mice with different doses of extract of *P.* *pavonica* and *J.* *rubens,* results show a minor decrease in the albumin concentration in EAC mice post i.p. inoculation with *P.* *pavonica* and *J.* *rubens* extracts (Fig. 8). Insignificantly decreased the albumin level in serum in all groups, compared to the EAC mice and Cisplatin^*®*^-treated EAC mice. Remarkably, a high dose of *P.* *pavonica* extract significantly diminished the serum albumin level compared to naïve EAC mice (Fig. 8).

**Fig. 9:**
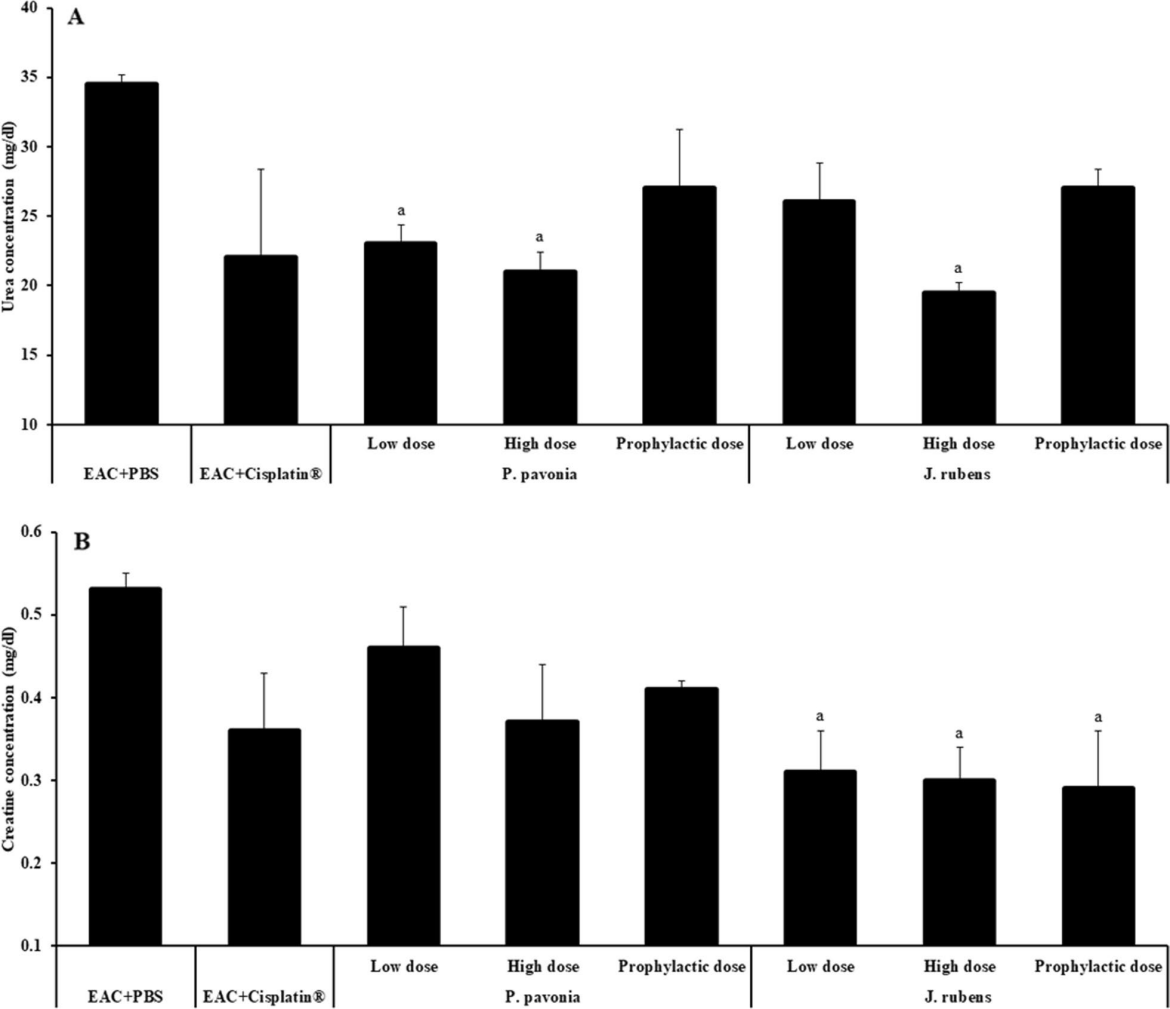
The effects *P.* *pavonica* and *J.* *rubens* extracts on kidney function in EAC–bearing mice: Mice (n = 10) were i.p inoculated with *P.* *pavonica* at low dose (1.3 μg/mouse), high dose (2.5 μg/mouse) or prophylactic dose (2.5 μg/mouse), *J.* *rubens* at low dose (1.2 μg/mouse), high dose (2.3 μg/mouse) or prophylactic dose (2.3 μg/mouse), Cisplatin^®^ (10 μg/mouse) or phosphate buffer saline (PBS). Data were represented as mean ± SD. The difference between groups was considered statistically significant at *P* < 0.05. ^a^Statistically significant vs. EAC mice

Treatment with *P.* *pavonia* extract at low, high and prophylactic doses (1.3 μg/mouse, 2.5 μg/mouse, and 2.5 μg/mouse, respectively) and *J.* *rubens* extract at high dose (2.3 μg/mouse) had a significant decrease in urea concentration compared to naïve EAC mice. Meanwhile, i.p inoculation with *P.* *pavonica* extract at prophylactic doses (2.5 μg/mouse) and *J.* *rubens* extract at a low and prophylactic dose (1.2 μg/mouse and 2.3 μg/mouse, respectively) resulted in a nonsignificant increase in urea concentration compared to Cisplatin®-treated EAC mice (Fig. 9). Interperitoneal inoculation with *P.* *pavonica* extract at low, high and prophylactic doses (1.3 μg/mouse, 2.5 μg/mouse, and 2.5 μg/mouse, respectively) and *J.* *rubens* extract at low, high and prophylactic doses (1.2 μg/mouse, 2.3 μg/mouse and 2.3 μg/mouse, respectively) led to a nonsignificant decrease in creatinine concentration compared to naïve EAC mice. (Fig. 9). High and prophylactic doses of *J.* *rubens* extract i.p. inoculated into EAC mice resulted in a significant decrease in creatinine level compared to Cisplatin^®^-treated EAC mice (Fig. 9).

**Fig. 10:**
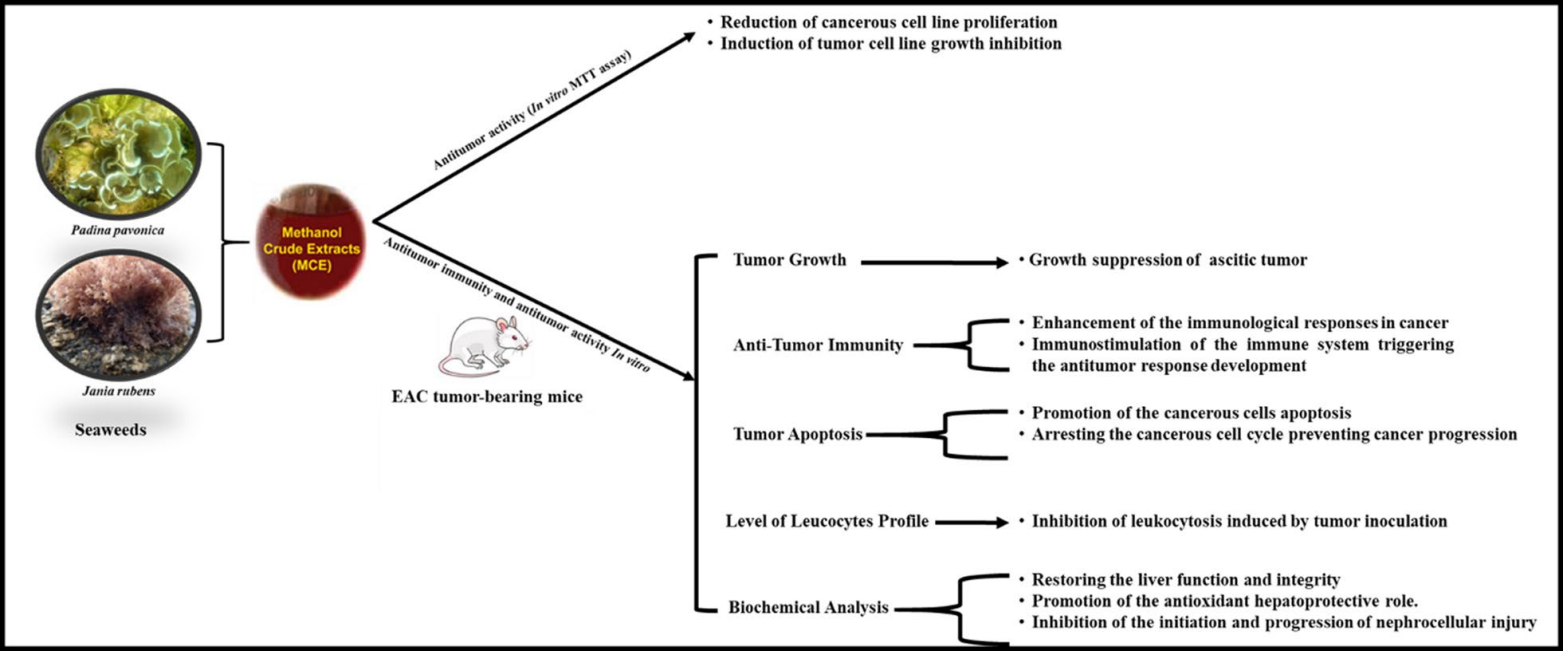
Schematic diagram of the overall results of the manuscript

Overall, our results collectively indicated that in vitro antitumor efficacy investigations of *P.* *pavonica,* *J.* *rubens* *extracts* *showed* *a* reduction in the proliferation and an induction in the growth inhibition of human liver cancer Hep-G2 cell line. On the other hand, in vivo investigations of the efficacy of *P.* *pavonica,* *J.* *rubens* *extracts* *in* antitumor immunity, tumor growth, tumor apoptosis and biochemical analysis indicated a growth suppression of ascitic tumor cells, enhancement of the immunological responses in cancer, immunostimulation of the immune system triggering the antitumor response development, promotion of the cancerous cells apoptosis, arresting the cancerous cell cycle preventing cancer progression, inhibition of leukocytosis induced by tumor inoculation, restoring the liver function and integrity, promotion of the antioxidant hepatoprotective role and inhibition of the initiation and progression of nephrocellular injury (Fig. 10).

## Discussion

### In vitro *antitumor activity*

The current research was planned to detect the potential immunomodulation and antitumor efficacy of the extracts of *P.* *pavonica* and *J.* *rubens* inhabiting the Egyptian water. Our overall data has succeeded to present the extracts derived from *P.* *pavonica* and *J.* *rubens* as novel immune regulators and a promising anticancer agent against Ehrlich ascites breast carcinoma (EAC). Cancer is the world's most serious health concern, and the best treatment is cancer chemotherapy, which is cytotoxic to normal cells as well. Seaweeds are new, inexpensive, healthy, and efficient anticancer agents with minimal side effects on normal cells [38–40]. Seaweed-derived extracts, such as *P.* *pavonica* and *J.* *rubens*, have been described as immunomodulatory and anticancer drugs, immunological regulators stimulating immune cells, enhancing immune function, and acting as adjuvants in cancer immunotherapy [21–24].

In a dose-dependent manner, treatment of EAC cells with *P.* *pavonica* and *J.* *rubens* extracts resulted in significant reductions in the number and survival of EAC tumor cells in an in vitro investigation. Regardless of differences in the chemical composition of the *P.* *pavonica* and *J.* *rubens* extracts; both induced direct antitumor effects, indicating that the biological products of genus *Padina* and *Jania* have potent antitumor effects. Despite the antitumor effects of these two agents were not the same as those of cisplatin^®^ (reference drug), our data had shown the promising antineoplastic effects of *P.* *pavonica* and *J.* *rubens* extracts especially at high doses (1000 μg/mL) in vitro. It is well known that cancer cells possess a high proliferative rate and low apoptotic rate -opposite to normal cells and the antitumor potential of conventional chemotherapeutic drugs such as Cisplatin^®^ mostly mediated at least in part, by increasing apoptosis, and decreasing DNA proliferation of rapidly dividing cells [41, 42].

We found that *P.* *pavonica* and *J.* *rubens* extracts caused an elevation in EAC cell apoptosis Accompanied by a decrease in cell proliferation post i.p. treatment of EAC tumor cells for 24 h in vitro. In vitro studies also showed that the *J.* *rubens* extract had a higher apoptotic impact than *T.* *atomaria*, *P.* *pavonica*, and *E.* *elongata* extracts in enhancing the rate of EAC apoptosis, with IC_50_ values of 475.1, 580, 613, and 630.9 g/mL, respectively. Previously, it was reported that polysaccharides crude extracts of *P.* *pavonica,* *J.* *rubens,* and *Corallina* *officinalis* (Rhodophyta) significantly inhibited the human breast MCF7 and colon cancer CoCa_2_, liver cancer cell line HePG2 cell lines proliferation in vitro when compared to the reference drug doxorubicin [43, 44]. The biological and antitumor activity detected in the tested seaweed extracts may be associated with the presence of oxygenated sterols and diterpenes [45], even though these should be further studied. The differences in the antitumor potentials between *P.* *pavonica* extracts and *J.* *rubens* extracts may be due to specific variations, environmental habitats, the used concentrations, or other factors that need further studies.

### *Antitumor effects of different doses of seaweeds extracts on the host tumor growth* in vivo

In vitro investigations of growth suppression of EAC tumor cells are consistent and strongly suggest that *P.* *pavonica* and *J.* *rubens* extracts may have anticancer characteristics. Next, we tended to evaluate whether the direct immunomodulation and antitumor efficacy of the *P.* *pavonica* and *J.* *rubens* extracts can also be observed in vivo. In comparison to naïve EAC mice and Cisplatin^®^-treated EAC mice, i.p. treatment of EAC-bearing mice with *P.* *pavoncia* extract at low, high, and prophylactic doses (1.3 g/mouse, 2.5 g/mouse, and 2.5 g/mouse, correspondingly) or *J.* *rubens* extract at low, high, and prophylactic doses (1.2 μg/mouse, 2.3 μg/mouse and 2.3 μg/mouse, respectively) have resulted in a considerable reduction in the number of ascetic EAC tumor cells. Previously, *Ulva* *rigida* (Chlorophyta) extracts significantly decrease the tumor's developmental size, *J.* *rubens* extract at 250 mg/kg and 500 mg/kg arrest the cell proliferation and tumor cell growth, and *Kappaphycus* *alvarezii* (formerly *Eucheuma* *cottonii*) (Rhodophyta) extract suppressed tumor growth and reduce mammary gland tumor development in rats [46–48]. Bioactive compounds extracted from different types of seaweeds exhibited anticancer potentials against human cancers [16]. So that seaweed extracts could be a promising and safe anticancer candidate for human cancers [49].

### Changes in the level of leucocytes profile

In addition to the direct antitumor effect exerted by both extracts, we hypothesized that both extracts may exert an immunomodulatory effect that could indirectly potentiate their antitumor efficacy in vivo. We investigated the systemic immunomodulatory potentials of tumor inoculation and the ameliorative effect of *P.* *pavoncia* and *J.* *rubens* extracts. The i.p. inoculation of EAC tumor cells induced significant leukocytosis. Furthermore, an elevated leukocyte count linked to the onset of cancer has been found as one of the predictors of an increased risk of venous thromboembolism (VTE) in cancer patients. Thrombosis is the second greatest cause of cancer-related mortality [50]. Interestingly, we explored that treatment of EAC mice with either Cisplatin^®^ or *P.* *pavonica* extract or *J.* *rubens* extract significantly increased the leukocytosis induced by tumor inoculation. Moreover, both *P.* *pavonica* and *J.* *rubens* extracts displayed a more powerful efficacy than Cisplatin^®^ in the inhibition of leukocytosis. Accordingly, this revealed that *P.* *pavonia* and *J.* *rubens* extracts may be potent tools for not only cancer treatment but also, for inhibition of thrombosis. Hence, they will help better the prognosis for cancer patients.

We hypothesized that i.p. inoculation of EAC mice with *P.* *pavonica* and *J.* *rubens* extracts did not increase the total number of WBCs but elevated their flow to the tumor site. The migratory capacity of leukocytes is crucial to their role as defensive cells implicating their role in the progression and spread of tumors. Different leukocyte populations, including neutrophils, dendritic cells, macrophages, eosinophils, mast cells, and lymphocytes, are known to have a role in tumor-associated inflammation [51]. Our results revealed that i.p. inoculation with *P.* *pavonica* and *J.* *rubens* extracts managed to decrease neutrophilia induced by tumor inoculation. Neutrophilia is one of the hematological findings related to poor prognosis in human metastatic melanoma, pancreatic carcinoma, and renal carcinoma [52, 53].

Remarkably, treatment of EAC mice with *P.* *pavonica* *and* *J.* *rubens* extracts at low, high, and prophylactic doses induced a slight increase in the lymphocytes relative number compared to naïve EAC mice and Cisplatin®-treated EAC mice. Unlike neutrophils, the increased lymphocytic count is considered a good prognostic candidate in diverse cancers [54].

Monocyte subsets are immune cells that have recently been identified as key regulators of cancer growth and development. Phagocytosis, tumoricidal mediator secretion, angiogenesis, extracellular matrix remodeling, lymphocyte recruitment, and tumor-associated macrophages differentiation and dendritic cells are all functions performed by different monocyte subsets that associate to the antitumoral immunity [55]. Intraperitoneal treatment of EAC mice with *P.* *pavonica* at high or prophylactic doses and *J.* *rubens* extracts at low, high or prophylactic doses has successfully decreased the monocytes relative number compared to naïve EAC mice and Cisplatin^®^-treated mice. Meanwhile, a low dose of *P.* *pavonica* extract seemed to increase the relative number of monocytes compared to naïve EAC mice. Such data could be consistent with the associated neutropenia induced by EAC tumor cells treatment. As, withdrawal of neutrophils numbers is accompanied by their transformation into other myeloid cells including MDSCs [56].

### Flow cytometry analysis

Algal extracts have been widely used in cancer immunotherapy due to their properties as an initiator and modulator of immune responses, and their vaccinations appear to be safe and enhance immunological responses in cancer treatment as well as long-term clinical remissions, but there are some issues that need to be addressed before they can be used clinically. According to flow cytometric analysis of phenotypic expression of immune lymphocyte cells subset surface markers, treatment of mice with *P.* *pavonica* and *J.* *rubens* extracts at low, high, and prophylactic doses showed putative immune-modulatory effects on the expression and proliferative response of spleen lymphocyte subpopulations; CD4 + T, CD8 + T, and NK cells.

*P.* *pavonica* and *J.* *rubens* administration increased the percentage of lymphocyte subpopulations; CD4^+^ T cells, CD8^+^ T cells, and CD335 cells and lymphocyte proliferative responses that were stimulatory at all tested doses, and there was a dose-dependent increase in the proliferative activity of CD4 + T-cells, CD8 + T cells, and NK cells in EAC-bearing mice comparing to naïve EAC mice and Cisplatin^®-^tread EAC mice.

More specifically, i.p. inoculation of EAC mice with *P.* *pavonica* and *J.* *rubens* extracts induced an immune response favoring tumor cell death, tumor antigen cross-presentation in vivo, and the production of cytokines favoring homeostatic proliferation and/or ablation of immunosuppression mechanisms [57]. The change in the ratio of CD4^+^T-cell, CD8^+^T-cell, may be attributed to the migration of immune cells populations to the developed tumor, leading to a significant reduction of the same population in the spleen.

Natural killer (NK) cells are an innate immune system subpopulation of lymphocytes that play a key role in the host defense against tumor growth and infectious pathogens [58]. NK cells can detect and kill a broad variety of abnormal cells, like tumor cells, without damaging healthy cells [59]. The same findings as polysaccharide from *Laminaria* *digitat* (Phaeophyceae) activated immune systems, B and helper T lymphocytes were evaluated in the literature [60]. Later, the fucoidan isolated from *Sargassum* sp. and *Fucus* *vesiculosus* (Phaeophyceae) stimulates natural killer cell activity in vivo in noncancerous mice [61]. Data from in vivo studies observed inhibitory activity of cancer, which could be attributed in part to increased innate and specific immunity [62]. Fucoidan from *Undaria* *pinnatifida* (Phaeophyceae) sporophyll increased survival in P-388 tumor-bearing mice, which was linked to the enhancement of natural killer (NK) lymphocyte activity and increased interferon-gamma production by T cells [29]. *Gracilariopsis* *lemaneiformis* (formerly *Gracilaria* *lemaneiformis*) (Rhodophyta) sulfated polysaccharides prevented tumor growth; increased CD8^+^ T cells, splenocyte proliferation, macrophage phagocytosis [63, 64].

Our results show that one of the antitumor mechanisms of the extract of *P.* *pavonica* and *J.* *rubens* may be to mediate an increase in T-lymphocytes subsets of helper (CD4^+^ T-cell), cytotoxic (CD8^+^ T-cell), and NK cells in the spleen that resulted in the production of immunostimulation of the immune system triggering the antitumor response development.

Seaweeds-derived products and their derivatives possess antitumor properties via modulating immune mechanism and a variety of cellular and molecular mechanisms, inducing apoptosis, cell cycle progression blocking, modulating transduction signal pathways, preventing cancer cell invasion, and metastasis, stopping angiogenesis and antioxidant activity [65–71].

### Assessment of apoptosis by flow cytometry

In the current study, we aimed to analyze the potentiation of both extracts on the apoptosis rate and proliferative rate of EAC tumor cells by Annexin V-FITC Apoptosis assay using flow cytometry, which targets the loss of integrity of the plasma membrane [72, 73]. It is well known that cancerous cells have a high proliferative degree and low apoptotic rate—contradictory to normal cells. The antitumor efficacy of conventional chemotherapeutic agent such as Cisplatin^®^ is generally mediated at least in part by elevating apoptosis rate and reducing DNA proliferation of rapidly dividing cells [41, 42].

Interestingly, *P.* *pavonica* and *J.* *rubens* extracts at all tested doses increased the percentages of apoptosis in a dose-dependent manner as high doses increase the percentages of apoptosis compared to low ones. Besides, analysis of the cell cycle revealed that the *P.* *pavonica* extract was of a more potent effect than *J.* *rubens* extract in increasing apoptosis of EAC cells in vivo. As it successfully increased EAC apoptosis even more than the therapeutic dose of Cisplatin^®^. It is recommended a strongly related activity of *P.* *pavonica,* and *J.* *rubens* extracts to develop an antitumor response in a tumor-bearing host.

Our data revealed that i.p. inoculation of EAC mice with *P.* *pavonica* and *J.* *rubens* extracts at low, high, and prophylactic doses resulted in an elevation in EAC cell apoptosis accompanied by a decrease in cell proliferation. Additionally, in vivo data indicated that the *P.* *pavonica* extract was of a more apoptotic effect than *J.* *rubens* extracts in increasing the rate of EAC apoptosis. The apoptosis mode of action may vary among different anticancer bioactive compounds, and the main anticancer mechanism of the *P.* *pavonica* and *J.* *rubens* extracts is the apoptosis induction [74]. Several other anticancer studies of sulfated polysaccharides from *Padina* *pavonica* and *Jania* *rubens* [43, 75] were published, with identical findings for both qualitative and quantitative apoptosis evaluation.

### Liver and kidney functions

Finally, serum liver transaminases (ALT and AST) and albumin are tested to rule out hepatocellular injury and hepatotoxicity as a side effect of therapy. Their increases in serum are a cellular outflow marker and a loss of functional integrity of hepatocyte cell membranes [76, 77]. We also investigate the serum kidney creatinine and urea, the most widely used biomarkers for determining the amount of nephrocellular damage, to rule out nephrocellular damage and nephrotoxicity caused by chemical stressors [78, 79].

Our data revealed that i.p. inoculation of *J.* *rubens* extracts at low, high or prophylactic doses, but not *P.* *pavonica* extract doses, has successfully decreased the level of serum AST, ALT, and albumin and showed a significant effect in restoring their levels to be very close to the naïve mice comparing to naïve EAC mice and Cisplatin^®^-treated EAC mice, indicating restoring liver function and integrity and promoting the antioxidant hepatoprotective role of *J.* *rubens* extracts [80]. A previous study reported that treatment of experimental animals with extract of *J.* *rubens*, *Sargassum* *subrepandum* (Phaeophyceae)*,* and *Ulva* *lactuca* (Chlorophyta) maintained liver enzymes in the range of the normal levels compared to the naïve tumor group. Therefore, methanol extract of *J.* *rubens* may be promising hepatoprotective agents that could synergistically inhibit the process of hepatocellular injury initiation and progress in combination with its antioxidant activity [81].

Hepatotoxicity may be induced by a high dose of Cisplatin^®^ [82]. One of the most significant pathways implicated in Cisplatin^®^ toxicity is oxidative stress and may be the primary source of Cisplatin^®^-induced toxicity attributable to decreased GSH glutathione depletion [83]. Transaminases are the most important biomarkers specifically involved in causing cell damage and toxicity, as they are released into circulation after cell damage.

Importantly, our results revealed that i.p. inoculation of *J.* *rubens* extracts at low, high or prophylactic doses, but not *P.* *pavonica* extract doses, has successfully decreased the concentrations of serum creatinine and urea and reported a significant effect in restoring their concentrations to be close to the naïve mice comparing to naïve EAC mice and Cisplatin^®^-treated EAC mice. As a result, methanol extract of *J.* *rubens* may be promising nephroprotective agents that could synergistically inhibit the initiation and progression of nephrocellular injury in combination with its antioxidant activity. This effect could be due to its antioxidant activities and active antioxidant constituents.

Nephrotoxicity is Cisplatin's main dose-limiting side effect [84]. Cisplatin^®^, as well as direct cellular toxicity, triggers an inflammatory cascade where neutrophils may induce kidney injury via different pathways such as the development of proinflammatory mediators, reactive species, proteases, and other products that may provoke tubular damage resulting in acute kidney injury [85].

## Conclusion

Further studies are needed to isolate the therapeutic bioactive components of *P.* *pavonica* and *J.* *rubens*, identify the underlying mechanisms of their antitumor immunity and antitumor cytotoxic effects, investigate their immunomodulatory effect that could indirectly potentiate their antitumor efficacy, and their ameliorative effects on tumor inoculation. Overall, both extracts of *P.* *pavonica* and *J.* *rubens* possess potential antitumor and antitumor immunological effects with less toxicity, opening new approaches for further studies of the chemical and biological mechanisms behind these effects.

## Data Availability

The datasets generated and analyzed during the current study are available from Eman Bases.

## Abbreviations

Hep-G2: Human hepatoma G2 cells
MTT: (3-(4,5-Dimethylthiazol-2-yl)
EAC: Ehrlich ascites carcinoma
WBC: White blood cells
MCF-7: Acronym of Michigan Cancer Foundation-7
IC_50_: 50% Inhibition concentration
VEGF: Vascular endothelial growth factor
NK: Natural killer
FBS: Fetal bovine serum
RPMI-1640: Roswell Park Memorial Institute Medium-1640
ACK: Ammonium-chloride-potassium
DMSO: Dimethyl sulfoxide
LD_50_: 50% Lethal dose
TBRI: Theodor Bilharz Research Institute
PB: Peripheral blood
ANOVA: A one-way analysis of variance
SD: Standard deviation
